# Characteristics and outcomes of patients with COVID-19 admitted to the ICU in a university hospital in São Paulo, Brazil - study protocol

**DOI:** 10.6061/clinics/2020/e2294

**Published:** 2020-08-21

**Authors:** Juliana C. Ferreira, Yeh-Li Ho, Bruno A.M.P. Besen, Luiz M.S. Malbuisson, Leandro U. Taniguchi, Pedro V. Mendes, Eduardo L.V. Costa, Marcelo Park, Renato Daltro-Oliveira, Roberta M.L. Roepke, João M. Silva, Maria José C. Carmona, Carlos Roberto Ribeiro Carvalho, EPICCoV Study Group, Adriana Hirota, Alberto Kendy Kanasiro, Alessandra Crescenzi, Amanda Coelho Fernandes, Anna Miethke-Morais, Arthur Petrillo Bellintani, Artur Ribeiro Canasiro, Bárbara Vieira Carneiro, Beatriz Keiko Zanbon, Bernardo Pinheiro De Senna Nogueira Batista, Bianca Ruiz Nicolao, Bruno Adler Maccagnan Pinheiro Besen, Bruno Biselli, Bruno Rocha De Macedo, Caio Machado Gomes De Toledo, Carlos Eduardo Pompilio, Carlos Roberto Ribeiro De Carvalho, Caroline Gomes Mol, Cassio Stipanich, Caue Gasparotto Bueno, Cibele Garzillo, Clarice Tanaka, Daniel Neves Forte, Daniel Joelsons, Daniele Robira, Eduardo Leite Vieira Costa, Elson Mendes Da Silva, Fabiane Aliotti Regalio, Gabriela Cardoso Segura, Gustavo Brasil Marcelino, Giulia Sefrin Louro, Yeh-Li Ho, Isabela Argollo Ferreira, Jeison de Oliveira Gois, Joao Manoel Da Silva, Jose Otto Reusing, Julia Fray Ribeiro, Juliana Carvalho Ferreira, Karine Vusberg Galleti, Katia Regina Silva, Larissa Padrao Isensee, Larissa dos Santos Oliveira, Leandro Utino Taniguchi, Leila Suemi Letaif, Lígia Trombetta Lima, Lucas Yongsoo Park, Lucas Chaves, Luciana Cassimiro Nobrega, Luciana Haddad, Ludhmila Hajjar, Luiz Marcelo Malbouisson, Manuela Cristina Adsuara Pandolfi, Marcelo Park, Maria José Carvalho Carmona, Maria Castilho Prandini H De Andrade, Mariana Moreira Santos, Matheus Pereira Bateloche, Mayra Akimi Suiama, Mayron Faria de Oliveira, Mayson Laercio Sousa, Michelle Louvaes, Natassja Huemer, Pedro Mendes, Paulo Ricardo Gessolo Lins, Pedro Gaspar Dos Santos, Pedro Ferreira Paiva Moreira, Renata Mello Guazzelli, Renato Batista Dos Reis, Renato Daltro De Oliveira, Roberta Muriel Longo Roepke, Rodolpho Augusto De Moura Pedro, Rodrigo Kondo, Samia Zahi Rached, Sergio Roberto Silveira Da Fonseca, Thais Sousa Borges, Thalissa Ferreira, Vilson Cobello, Vivian Vieira Tenório Sales, Willaby Serafim Cassa Ferreira

**Affiliations:** I Divisao de Pneumologia, Instituto do Coracao (InCor), Hospital das Clinicas HCFMUSP, Faculdade de Medicina, Universidade de Sao Paulo, Sao Paulo, SP, BR.; II Unidade de Terapia Intensiva, AC Camargo Cancer Center, Sao Paulo, SP, BR.; III Divisao de Molestias Infecciosas, Hospital das Clinicas HCFMUSP, Faculdade de Medicina, Universidade de Sao Paulo, Sao Paulo, SP, BR.; IV UTI Clinica, Disciplina de Emergencias Clinicas, Departamento de Clinica Medica, Hospital das Clinicas HCFMUSP, Faculdade de Medicina, Universidade de Sao Paulo, Sao Paulo, SP, BR.; V Divisao de Anestesia, Hospital das Clinicas HCFMUSP, Faculdade de Medicina, Universidade de Sao Paulo, Sao Paulo, SP, BR.; VI UTI Emergencias Cirurgicas e Trauma, Departamento de Cirurgia, Hospital das Clinicas HCFMUSP, Faculdade de Medicina, Universidade de Sao Paulo, Sao Paulo, SP, BR.

**Keywords:** Ventilation, Artificial, Severe Acute Respiratory Syndrome, Intensive Care Units, SARS Virus, COVID-19

## Abstract

**OBJECTIVES:**

We designed a cohort study to describe characteristics and outcomes of patients with coronavirus disease (COVID-19) admitted to the intensive care unit (ICU) in the largest public hospital in Sao Paulo, Brazil, as Latin America becomes the epicenter of the pandemic.

**METHODS:**

This is the protocol for a study being conducted at an academic hospital in Brazil with 300 adult ICU beds dedicated to COVID-19 patients. We will include adult patients admitted to the ICU with suspected or confirmed COVID-19 during the study period. The main outcome is ICU survival at 28 days. Data will be collected prospectively and retrospectively by trained investigators from the hospital’s electronic medical records, using an electronic data capture tool. We will collect data on demographics, comorbidities, severity of disease, and laboratorial test results at admission. Information on the need for advanced life support and ventilator parameters will be collected during ICU stay. Patients will be followed up for 28 days in the ICU and 60 days in the hospital. We will plot Kaplan-Meier curves to estimate ICU and hospital survival and perform survival analysis using the Cox proportional hazards model to identify the main risk factors for mortality. ClinicalTrials.gov: NCT04378582.

**RESULTS:**

We expect to include a large sample of patients with COVID-19 admitted to the ICU and to be able to provide data on admission characteristics, use of advanced life support, ICU survival at 28 days, and hospital survival at 60 days.

**CONCLUSIONS:**

This study will provide epidemiological data about critically ill patients with COVID-19 in Brazil, which could inform health policy and resource allocation in low- and middle-income countries.

## INTRODUCTION

In December 2019, an outbreak of severe acute respiratory syndrome (SARS) attributed to a new coronavirus, namely, severe acute respiratory syndrome coronavirus-2 (SARS-CoV-2), was identified in the Wuhan region of China (1-3). The disease caused by the virus, called coronavirus disease (COVID-19), is characterized by a flu-like syndrome, with symptoms such as fever, cough, myalgia, and gastrointestinal symptoms (4). Most cases are mild, some may be asymptomatic, but approximately 15% of patients have a more severe presentation, and approximately 5% are critical (5). The acute respiratory failure among critically ill patients with COVID-19 is unique in its presentation: difficult-to-treat hypoxemia, clotting disorders, renal failure, and changes in immunity and inflammatory phenomena (6-8) all pose challenges to the management of these patients.

The virus spread from China to Europe and then the Americas, causing more than 20 million cases and hundreds of thousands of deaths as of August 15^th^ (9). Brazil is one of the countries with the highest number of cases as well as deaths, and Sao Paulo is currently the state with highest number of cases in the country (10), with over 26,000 deaths by mid-August.

Reports of the most important risks factors and mortality rates have become available (11-17), but as Latin America emerges as the new hotspot of the pandemic, there are no epidemiological data on COVID-19 or any epidemic of this magnitude in low- and middle-income countries (LMICs) that could help predict the impact of the new disease on its health system. The burden of critical illness is higher in LMICs (18), and the mortality of patients on mechanical ventilation in Brazil is high (19). Given the differences in the age distribution of the population, intensive care unit (ICU) capacity, lower availability of diagnostic testing for the new virus, difficulties in the implementation of public measures to mitigate virus spread in highly populated cities, and delayed access to health care, the burden of COVID-19 tends to be higher in LMICs than in high-income countries (HIC).

Knowing the characteristics of critically ill patients with COVID-19 and their clinical outcomes in LMICs is extremely important to inform clinical decision-making and public health management in this setting. Therefore, we designed an observational study to describe the characteristics and outcomes of patients admitted to the ICUs of the largest public hospital in Sao Paulo during the peak of the COVID-19 pandemic in Brazil.

## MATERIALS AND METHODS

### Study design and location

This is a cohort study conducted at Hospital das Clínicas from University of Sao Paulo Medical School. It is the largest academic, tertiary, university-affiliated hospital in Brazil with 300 adult ICU beds dedicated exclusively to the care of COVID-19 patients during the peak of the pandemic in Sao Paulo.

The Research Ethics Committee of Hospital das Clínicas da Universidade de Sao Paulo approved the study protocol (number 31382620.0.0000.0068), and the study was registered in a public database (clinicaltrials.gov, NCT04378582). The requirement for informed consent was waived because of the observational nature of the study.

From March 13 to April 16, 200 previously functioning ICU beds were converted into COVID-19-dedicated ICU beds. In the following 45 days, operating rooms and several hospital wards were repurposed to accommodate 100 additional COVID-19-ICU beds. Physicians, nurses, and respiratory therapists were hired to constitute new ICU teams dedicated to patients occupying these additional beds. New staff were trained and distributed within experienced ICU teams.

The study is observational, and patient care was carried out following institutional protocols developed specifically for COVID-19 patients. The protocol recommends routine use of deep venous thrombosis prophylaxis and systemic antibiotics if bacterial coinfection is suspected. Specific drugs for treating SARS-CoV-2 were not recommended but could be administered at the discretion of the attending physician. The study also included specific protocols for the ventilatory management of respiratory failure and for sedation management throughout ICU stay. Other critical care standards were not changed during the pandemic.

### Study population

**Figure 1:**
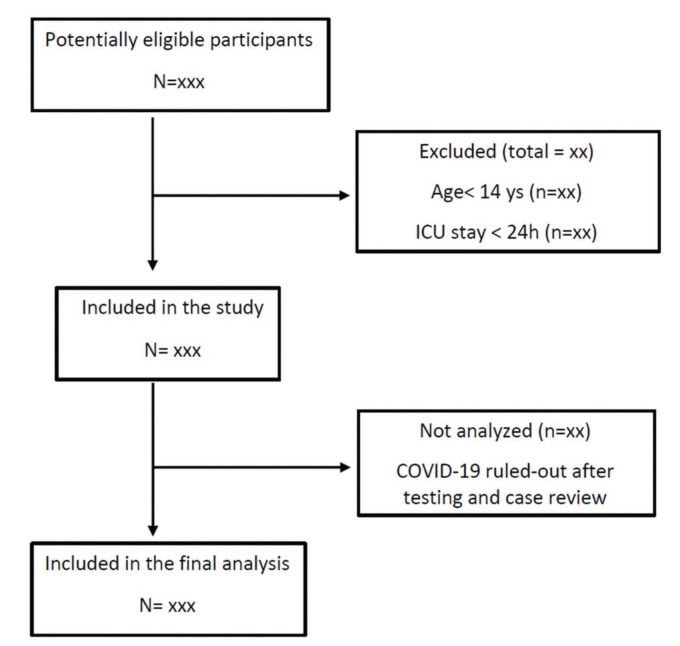
Expected flow of potentially eligible participants in the study.

We will include all consecutive adult patients with suspected or confirmed COVID-19 admitted to the ICUs between March 30 and June 30, 2020 with an expected ICU stay longer than 24 hours (Figure 1). The only exclusion criterion is ICU stay shorter than 24 hours. Patients readmitted to the ICU during their hospital stay will be included in the study only at their first ICU admission. Patients will be classified as confirmed COVID-19, highly suspected COVID-19 without laboratory confirmation, and ruled-out COVID-19. Positive results on either an RT-PCR assay of nasal specimens, throat-swab specimens, or tracheal aspirate or a serologic (antibody) test will be used to confirm SARS-CoV-2 infection. Patients highly suspected of having an infection but with negative RT-PCR test results initially will be submitted to a second RT-PCR test and/or a serologic test after seven days of symptom onset. Patients with ruled-out SARS-CoV-2 infection will be transferred to another facility in the same institution and will be excluded from analysis.

### Outcomes

The main outcome is ICU survival at 28 days. Secondary outcomes include duration of mechanical ventilation, need for rescue therapies for severe hypoxemia, need for renal replacement therapy, ICU complications, and hospital survival at 60 days.

### Data collection, management, and quality control

**Figure 2:**
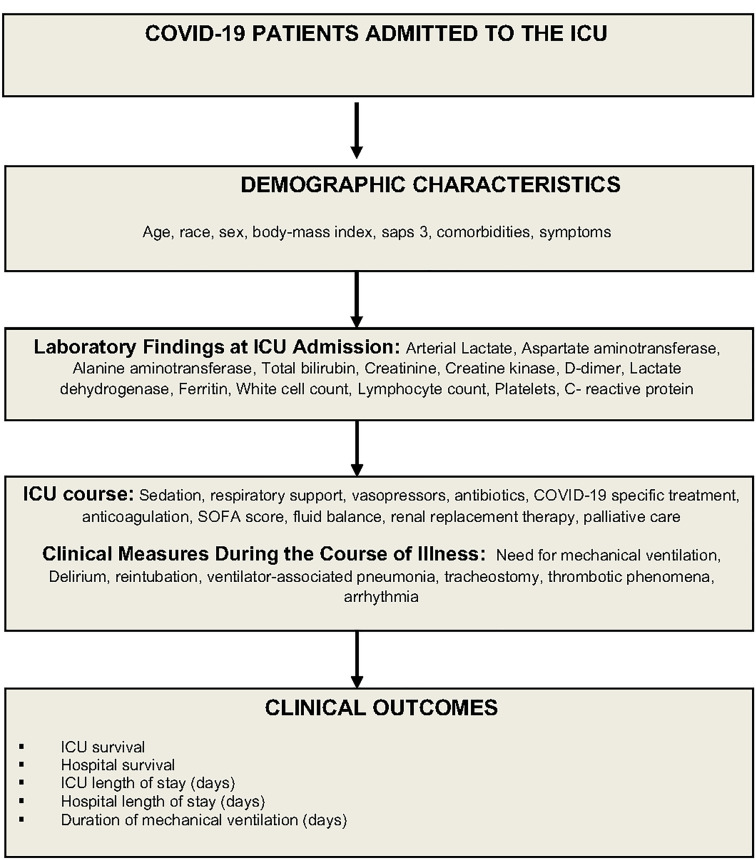
Overview of data collection and follow up in the study.

Data will be collected prospectively from study approval (May 6) to August 30, 2020 and retrospectively from March 30 to May 5, 2020. Trained data collectors will retrieve patient information from the hospital electronic medical records, and at the bedside (Figure 2). Study data will be collected and managed using a secure, web-based data collection platform (REDCap - Research Electronic Data Capture) (20,21). Data collected include demographic information, race, initial symptoms, comorbidities, outpatient medications, simplified acute physiology score (SAPS 3) (22,23), and laboratorial test results at admission (Table 1 and 2). We will also collect data on the Sequential Organ Failure Assessment score (SOFA) (24), need for oxygen support, mechanical ventilator parameters, use of specific interventions to treat SARS-CoV-2 infection, and decision to withhold or withdraw life support (Table 3). Patients will be followed up for 28 days, and data on exposures and outcomes, such as use of non-invasive ventilation, use of a high-flow nasal cannula, number of days on mechanical ventilation, need for renal replacement therapy, tracheostomy, occurrence of thromboembolic events, use of prone positioning or extracorporeal membrane oxygenation (ECMO) support, and ICU discharge status, will be collected (Table 4). We will follow up patients to register vital status at 60 days or at hospital discharge, whichever occurs first.

Quality control measures will include the use of REDCap-based structured data collection forms, training data collectors to ensure data completeness and consistency, and data management processes within the platform to deal with missing data, outliers, and data collection mistakes.

We will report study results in accordance to recommended guidelines for reporting observational studies, the Strengthening The Reporting of Observational Studies in Epidemiology (STROBE) (25), and the guidance from Pulmonary, Critical Care and Sleep journals on causal inference and prediction research (26,27).

### Statistical analysis plan

A sample size of 300 patients was initially anticipated. As the epidemic in Sao Paulo grew fast, the hospital included new ICU beds, and we revised the anticipated sample size to 500 patients. Given that the study carries no risks for participants and that the requirement for informed consent was waived by the IRB, we intend to collect data on all patients with COVID-19 admitted to the ICUs during the study period, a sample possibly larger than the anticipated sample size. Such a large sample would suffice to allow identification of predictors of survival.

Categorical variables will be expressed as counts and percentages, and continuous variables, as mean (standard deviation) or median (interquartile range), as appropriate. All hypothesis tests will be two-tailed, with a significance level of 0.05, and they will be performed using the R software (R Core Team, 2016, Vienna, Austria). We will build Kaplan-Meier curves to estimate 28-days survival. ICU survival will be defined as the time interval between ICU admission and patient death from any cause or ICU discharge. Time will be censored at 28 days for patients who are still alive. Hospital survival will be defined as the time interval between ICU admission and patient death from any cause or hospital discharge. Patients discharged home will be considered alive at day 28. We will perform survival analysis using the Cox proportional hazards model censored at 28 days to identify the main risk factors for ICU survival beyond expected associations, such as age, comorbidity burden, and severity of disease at ICU admission. Variables identified *a priori* as clinically relevant (Table 5) and additional variables with a *p*-value <0.20 in a univariate analysis will be evaluated as independent risk factors of poor prognosis in a multivariable Cox model. The order of inclusion of variables into Cox multivariable models will follow statistical significance criteria and clinical relevance. Alternative models will be chosen on the basis of information criteria and likelihood ratio-based tests. Wherever possible, continuous variables will be modeled to account for nonlinearity with splines or polynomials and avoiding dichotomization. Additivity will be addressed with interaction terms. The proportional hazards assumption will be tested using Schoenfeld residuals. We will use mixed models to analyze the impact of ventilatory variables collected during hospitalization on the main clinical outcomes. We will perform complete case analyses, without missing data. We will also perform a sensitivity analysis using multiple imputation.

## DISCUSSION

Our study will report characteristics and outcomes of all patients admitted to the ICU at the largest academic hospital in Brazil during the peak of the pandemic of COVID-19 in Sao Paulo. The hospital developed a preparedness plan that included grouping COVID-19 patients in a building dedicated exclusively for the care of these patients, creation of surge ICUs to increase ICU bed capacity, and hiring additional healthcare professionals to work exclusively in the COVID-19 ICUs. The actions were coordinated by a crisis committee composed of critical care specialists, pulmonologists, infectious disease specialists, nurses, respiratory specialists, pharmacists, engineers, and administrators, among others, who met daily to make shared decisions. Such an effort helped prevent shortage of personal protective equipment and important medications, facilitated the development of institutional protocols for patient care and infection control, and contributed to a rational distribution of limited resources with potential impact on patient outcomes (28). The results will allow for the assessment of clinical outcomes of critically ill patients that will be representative of a middle-income country in a highly strained environment caused by the pandemic. Overall healthcare inequity in the treatment of chronic comorbidities in the Brazilian healthcare system, delayed access to proper critical care, and heterogeneity in ICU within the hospital (surge *vs.* previously functioning ICUs) are some of the system-level characteristics that highlight the importance of this study to assess overall survival of COVID-19 patients in LMICs.

We will also be able to assess resource utilization beyond mechanical ventilation, such as renal replacement therapy and ICU length of stay, to characterize the burden of this surge of severe respiratory failure patients to our hospital and healthcare system. In the main manuscript, both characterization of the cohort and the risk factors for increased mortality will be assessed and compared to the current literature. Our main benchmark for ICU and hospital survival is the result published regularly by the Intensive Care National Audit & Research Centre (ICNARC), that is, 35%-45% hospital mortality among patients receiving mechanical ventilation (29), which represents the outcomes of a universal healthcare system in a HIC.

In addition, the study will provide answers to many ancillary research questions:

- Rate of use and success rate of non-invasive ventilation and high-flow nasal cannula use to treat respiratory failure and of commonly used strategies for refractory respiratory failure, including inhaled nitric oxide, prone positioning, and ECMO.
- Prediction models of hospital mortality specific for COVID-19.
- Characterization of subgroups of interest, such as oncologic and transplant patients.
- System-level research questions to compare outcomes and processes of care in “surge” ICUs and previously functioning ICUs, which would allow planning for future surges in critical care capacity.

In conclusion, we will be able to describe the characteristics and outcomes of a large sample of critically ill patients with COVID-19 admitted to a dedicated hospital in a LMIC, which could inform health policy and resource allocation and allow for many exploratory research questions to be answered.

### EPICCoV study investigators (in alphabetical order):

Adriana Hirota, Alberto Kendy Kanasiro, Alessandra Crescenzi, Amanda Coelho Fernandes, Anna Miethke-Morais; Arthur Petrillo Bellintani, Artur Ribeiro Canasiro, Bárbara Vieira Carneiro, Beatriz Keiko Zanbon, Bernardo Pinheiro De Senna Nogueira Batista, Bianca Ruiz Nicolao, Bruno Adler Maccagnan Pinheiro Besen, Bruno Biselli, Bruno Rocha De Macedo, Caio Machado Gomes De Toledo, Carlos Eduardo Pompilio, Carlos Roberto Ribeiro De Carvalho, Caroline Gomes Mol, Cassio Stipanich, Caue Gasparotto Bueno, Cibele Garzillo, Clarice Tanaka, Daniel Neves Forte, Daniel Joelsons, Daniele Robira, Eduardo Leite Vieira Costa, Elson Mendes Da Silva Júnior, Fabiane Aliotti Regalio, Gabriela Cardoso Segura, Gustavo Brasil Marcelino, Giulia Sefrin Louro, Yeh-Li Ho, Isabela Argollo Ferreira, Jeison de Oliveira Gois, Joao Manoel Da Silva Junior, Jose Otto Reusing Junior, Julia Fray Ribeiro, Juliana Carvalho Ferreira, Karine Vusberg Galleti, Katia Regina Silva, Larissa Padrao Isensee, Larissa dos Santos Oliveira, Leandro Utino Taniguchi, Leila Suemi Letaif, Lígia Trombetta Lima, Lucas Yongsoo Park, Lucas Chaves Netto, Luciana Cassimiro Nobrega, Luciana Haddad, Ludhmila Hajjar, Luiz Marcelo Malbouisson, Manuela Cristina Adsuara Pandolfi, Marcelo Park, Maria José Carvalho Carmona, Maria Castilho Prandini H De Andrade, Mariana Moreira Santos, Matheus Pereira Bateloche, Mayra Akimi Suiama, Mayron Faria de Oliveira, Mayson Laercio Sousa, Michelle Louvaes, Natassja Huemer, Pedro Mendes, Paulo Ricardo Gessolo Lins, Pedro Gaspar Dos Santos, Pedro Ferreira Paiva Moreira, Renata Mello Guazzelli, Renato Batista Dos Reis, Renato Daltro De Oliveira, Roberta Muriel Longo Roepke, Rodolpho Augusto De Moura Pedro, Rodrigo Kondo, Samia Zahi Rached, Sergio Roberto Silveira Da Fonseca, Thais Sousa Borges, Thalissa Ferreira, Vilson Cobello Junior, Vivian Vieira Tenório Sales, Willaby Serafim Cassa Ferreira. All investigators are from Hospital das Clinicas HCFMUSP, Faculdade de Medicina, Universidade de Sao Paulo, SP, BR.

## AUTHOR CONTRIBUTIONS

Ferreira JC, Besen BAP, Malbouisson LMS, Taniguchi LU, Ho YL, Mendes PV, Carmona MJC, and Carvalho CRR were responsible for the study concept and design. Ferreira JC, Besen BAP, Taniguchi LU, Mendes PV, Costa ELV, Park M, Daltro R, Roepke RML, Silva Jr JM and EPICCov site investigators were responsible for the acquisition, analysis, or interpretation of data. Ferreira JC, Besen BAP, Malbouisson LMS, Taniguchi LU, Ho YL, Mendes PV, Costa ELV, Park M, Daltro R, Roepke RML and Silva Jr JM were responsible for the manuscript drafting. Ferreira JC, Besen BAP, Malbouisson LMS, Taniguchi LU, Ho YL, Mendes PV, Costa ELV, Park M, Daltro R, Roepke RML, Silva Jr JM, Carmona MJC, Carvalho CRR and EPICCov site investigators were responsible for the critical revision of the manuscript for important intellectual content. Besen BAP, Costa ELV and Park M were responsible for the statistical analysis. Ferreira JC, Besen BAP, Ho YL and Malbouisson LMS were responsible for the study supervision.

## Figures and Tables

**Table 1:** Baseline characteristics at ICU admission.

Variable	All participants (n)
Age, years	mean (SD)
Race*	
White	No. (%)
Black	No. (%)
Brown	No. (%)
Asian	No. (%)
Indigenous	No. (%)
Other - not declared	No. (%)
Male sex	No. (%)
Body-mass index (kg/m^2^)	mean (SD)
SAPS 3	mean (SD)
SOFA	median (IQR)
Charlson’s comorbidities score	median (IQR)
Comorbidities	No. (%)
Asthma	No. (%)
Chronic Obstructive pulmonary disease	No. (%)
Cardiovascular disease	No. (%)
Hypertension	No. (%)
Diabetes	No. (%)
Chronic kidney disease of any stage	No. (%)
Cancer	No. (%)
Other	No. (%)
Symptoms	No. (%)
Loss of smell or taste	No. (%)
Fatigue	No. (%)
Headache	No. (%)
Altered level of consciousness	No. (%)
Rhinorrhea	No. (%)
Diarrhea	No. (%)
Shortness of breath	No. (%)
Sore throat	No. (%)
Fever	No. (%)
Myalgia	No. (%)
Cough	No. (%)
Duration of symptoms before admission, days	median (IQR)
Treatment before ICU admission	No (%)
Chloroquine or hydroxychloroquine	No (%)
Systemic corticosteroids	No (%)
Azithromycin	No (%)
Other antibiotics	No (%)

SAPS 3: Simplified acute physiology score; SOFA: Sequential Organ Failure Assessment score; *The categories represent the Brazilian official race classification.

**Table 2:** Laboratory findings at ICU admission.

Variable	patients (n)
Arterial lactate mg/dL	mean (SD)
Aspartate aminotransferase - U/L	mean (SD)
Alanine aminotransferase - U/L	mean (SD)
Total bilirubin - mg/dL	mean (SD)
Creatinine - mg/dL	mean (SD)
Creatine kinase - U/L	mean (SD)
D-dimer - ng/mL	mean (SD)
Lactate dehydrogenase - U/L	mean (SD)
Ferritin - ng/mL	mean (SD)
White blood cell count/mm^3^	mean (SD)
Lymphocyte count/mm^3^	mean (SD)
Platelet count/mm^3^	mean (SD)
C-reactive protein - mg/L	mean (SD)

**Table 3:** Patient management on day 1 after ICU admission.

Variables	All patients (n)
Sedation	No. (%)
Midazolam	No. (%)
Propofol	No. (%)
Fentanyl	No. (%)
Dexmedetomidine	No. (%)
Other	No. (%)
Respiratory support	
Oxygen catheter or mask	No. (%)
High-flow nasal cannula	No. (%)
Non-invasive ventilation	No. (%)
Invasive mechanical ventilation	No. (%)
Parameters during invasive ventilation (n=XXX)	
Tidal volume (mL)	mean (SD)
Minute volume (L/min)	mean (SD)
FIO_2_ (%)	mean (SD)
PaO_2_/FIO_2_	mean (SD)
PEEP (cmH_2_O)	mean (SD)
Plateau pressure (cmH_2_O)	mean (SD)
Driving pressure (cmH_2_O))	mean (SD)
pH	
PaCO_2_ (mmHg)	mean (SD)
Oxygen saturation (%)	mean (SD)
Rescue therapies	No. (%)
Prone position	No. (%)
Neuromuscular blockade	No. (%)
Inhaled pulmonary vasodilators	No. (%)
Extracorporeal membrane oxygenation	No. (%)
Recruitment maneuvers	No. (%)
PEEP titration by compliance or driving pressure	No. (%)
Vasopressors	No. (%)
Antibiotics	No. (%)
COVID-19 specific treatment	No. (%)
Chloroquine or hydroxychloroquine	No. (%)
Dexamethasone	No. (%)
Methylprednisolone	No. (%)
Hydrocortisone	No. (%)
Prednisone	No. (%)
Azithromycin	No. (%)
Lopinavir / ritonavir	No. (%)
Tocilizumab	No. (%)
Anticoagulation, No. (%)	No. (%)
Prophylactic dose	No. (%)
Full anticoagulation	No. (%)
Limitation of treatment	No. (%)

FIO_2_: fraction of inspired oxygen; PaO_2_: arterial partial pressure of oxygen; PEEP: positive pressure at end expiration.

**Table 4:** Clinical outcomes at ICU discharge.

Variable	Patients
Resource utilization	
Invasive mechanical ventilation	No. (%)
Prone positioning	No. (%)
Duration of Mechanical ventilation (days)	Median (IQR)
Use of NIV*	No. (%)
Use of high-flow nasal cannula*	No. (%)
Need for vasopressors	No. (%)
Renal replacement therapy	No. (%)
Tracheostomy	No. (%)
Clinical complications during ICU stay	
Delirium	No. (%)
Thrombotic phenomena	No. (%)
Ventilator-associated pneumonia	No. (%)
Cardiac arrythmias	No. (%)
Clinical outcomes	
ICU length of stay (days)	Median (IQR)
Hospital length of stay (days)	Median (IQR)
ICU mortality	No. (%)
Hospital mortality	No. (%)

*To avoid intubation or before intubation.

**Table 5:** Predictors of ICU survival at 28 days.

Variable	Survivors (n)	Non-survivors (n)	*p*-value*
Baseline characteristics			
Age,	mean (SD)	mean (SD)	
Male sex	No. (%)	No. (%)	
Charlson’s comorbidity score	Median (IQR)	Median (IQR)	
Obesity	No. (%)	No. (%)	
Acuity variables at admission#			
Need for vasopressors	No. (%)	No. (%)	
Invasive mechanical ventilation	No. (%)	No. (%)	
Glasgow coma scale	Median (IQR)	Median (IQR)	
Creatinine (mg/dL)	mean (SD)	mean (SD)	
Platelets	mean (SD)	mean (SD)	
Bilirubin (mg/dL)	mean (SD)	mean (SD)	
pH	mean (SD)	mean (SD)	
SAPS 3	mean (SD)	mean (SD)	
SOFA day 1	Median (IQR)	Median (IQR)	
Disease characteristics			
Time from symptom onset to ICU admission	Median (IQR)	Median (IQR)	
Number of days of intubation before ICU admission	Median (IQR)	Median (IQR)	
Other laboratorial results			
D-dimer (ng/mL)	mean (SD)	mean (SD)	
Lactate dehydrogenase (U/L)	mean (SD)	mean (SD)	
Lymphocyte count (cells/mm^3^)	mean (SD)	mean (SD)	
Arterial lactate (mg/dL)	mean (SD)	mean (SD)	
pH	mean (SD)	mean (SD)	

*p-value will come from univariate Cox proportional hazards model.

^#^During the first 24h of ICU stay.

## References

[1] Chan JF, Kok KH, Zhu Z, Chu H, To KK, Yuan S (2020). Genomic characterization of the 2019 novel human-pathogenic coronavirus isolated from a patient with atypical pneumonia after visiting Wuhan. Emerg Microbes Infect.

[2] Zhu N, Zhang D, Wang W, Li X, Yang B, Song J (2020). A Novel Coronavirus from Patients with Pneumonia in China, 2019. N Engl J Med.

[3] Wu F, Zhao S, Yu B, Chen YM, Wang W, Song ZG (2020). A new coronavirus associated with human respiratory disease in China. Nature.

[4] Chen N, Zhou M, Dong X, Qu J, Gong F, Han Y (2020). Epidemiological and clinical characteristics of 99 cases of 2019 novel coronavirus pneumonia in Wuhan, China: a descriptive study. Lancet.

[5] Zhou F, Yu T, Du R, Fan G, Liu Y, Liu Z (2020). Clinical course and risk factors for mortality of adult inpatients with COVID-19 in Wuhan, China: a retrospective cohort study. Lancet.

[6] Lillicrap D (2020). Disseminated intravascular coagulation in patients with 2019-nCoV pneumonia. J Thromb Haemost.

[7] Puelles VG, Lütgehetmann M, Lindenmeyer MT, Sperhake JP, Wong MN, Allweiss L (2020). Multiorgan and Renal Tropism of SARS-CoV-2. N Engl J Med.

[8] Qin C, Zhou L, Hu Z, Zhang S, Yang S, Tao Y (2020). Dysregulation of Immune Response in Patients With Coronavirus 2019 (COVID-19) in Wuhan, China. Clin Infect Dis.

[9] Johns Hopkins University COVID-19 Dashboard by the Center for Systems Science and Engineering (CSSE) at Johns Hopkins University (JHU).

[10] Ministério da Saúde Coronavírus Brasil. 2020.

[11] Wang Y, Lu X, Li Y, Chen H, Chen T, Su N (2020). Clinical Course and Outcomes of 344 Intensive Care Patients with COVID-19. Am J Respir Crit Care Med.

[12] Guan WJ, Ni Z, Hu Y, Liang WH, Ou CQ, He JX (2020). Clinical Characteristics of Coronavirus Disease 2019 in China. N Engl J Med.

[13] Grasselli G, Zangrillo A, Zanella A, Antonelli M, Cabrini L, Castelli A (2020). Baseline Characteristics and Outcomes of 1591 Patients Infected With SARS-CoV-2 Admitted to ICUs of the Lombardy Region, Italy. JAMA.

[14] Bhatraju PK, Ghassemieh BJ, Nichols M, Kim R, Jerome KR, Nalla AK (2020). Covid-19 in Critically Ill Patients in the Seattle Region — Case Series. N Engl J Med.

[15] Richardson S, Hirsch JS, Narasimhan M, Crawford JM, McGinn T, Davidson KW (2020). Presenting Characteristics, Comorbidities, and Outcomes Among 5700 Patients Hospitalized With COVID-19 in the New York City Area. JAMA.

[16] Arentz M, Yim E, Klaff L, Lokhandwala S, Riedo FX, Chong M (2020). Characteristics and Outcomes of 21 Critically Ill Patients With COVID-19 in Washington State. JAMA.

[17] Cummings MJ, Baldwin MR, Abrams D, Jacobson SD, Meyer BJ, Balough EM (2020). Epidemiology, clinical course, and outcomes of critically ill adults with COVID-19 in New York City: a prospective cohort study. Lancet.

[18] Diaz JV, Riviello ED, Papali A, Adhikari NKJ, Ferreira JC (2019). Global Critical Care: Moving Forward in Resource-Limited Settings. Ann Glob Health.

[19] Azevedo LC, Park M, Salluh JI, Rea-Neto A, Souza-Dantas VC, Varaschin P (2013). Clinical outcomes of patients requiring ventilatory support in Brazilian intensive care units: a multicenter, prospective, cohort study. Crit Care.

[20] Harris PA, Taylor R, Minor BL, Elliott V, Fernandez M, O’Neal L (2019). The REDCap consortium: Building an international community of software platform partners. J Biomed Inform.

[21] Harris PA, Taylor R, Thielke R, Payne J, Gonzalez N, Conde JG (2009). Research electronic data capture (REDCap)-a metadata-driven methodology and workflow process for providing translational research informatics support. J Biomed Inform.

[22] Moreno RP, Metnitz PG, Almeida E, Jordan B, Bauer P, Campos RA (2005). SAPS 3 - From evaluation of the patient to evaluation of the intensive care unit. Part 2: Development of a prognostic model for hospital mortality at ICU admission. Intensive Care Med.

[23] Metnitz PG, Moreno RP, Almeida E, Jordan B, Bauer P, Campos RA (2005). SAPS 3-From evaluation of the patient to evaluation of the intensive care unit. Part 1: Objectives, methods and cohort description. Intensive Care Med.

[24] Vincent JL, Moreno R, Takala J, Willatts S, De Mendonça A, Bruining H (1996). The SOFA (Sepsis-related Organ Failure Assessment) score to describe organ dysfunction/failure. On behalf of the Working Group on Sepsis-Related Problems of the European Society of Intensive Care Medicine. Intensive Care Med.

[25] von Elm E, Altman DG, Egger M, Pocock SJ, Gotzsche PC, Vandenbroucke JP, STROBE Initiative (2008). The Strengthening the Reporting of Observational Studies in Epidemiology (STROBE) statement: guidelines for reporting observational studies. J Clin Epidemiol.

[26] Lederer DJ, Bell SC, Branson RD, Chalmers JD, Marshall R, Maslove DM (2019). Control of Confounding and Reporting of Results in Causal Inference Studies. Guidance for Authors from Editors of Respiratory, Sleep, and Critical Care Journals. Ann Am Thorac Soc.

[27] Leisman DE, Harhay MO, Lederer DJ, Abramson M, Adjei AA, Bakker J (2020). Development and Reporting of Prediction Models: Guidance for Authors From Editors of Respiratory, Sleep, and Critical Care Journals. Crit Care Med.

[28] Pan A, Liu L, Wang C, Guo H, Hao X, Wang Q (2020). Association of Public Health Interventions With the Epidemiology of the COVID-19 Outbreak in Wuhan, China. JAMA.

[29] Intensive Care National Audit & Research Centre (2020). ICNARC report on COVID-19 in critical care.

